# Volunteers’ concerns about facial neuromuscular electrical stimulation

**DOI:** 10.1186/s40359-022-00827-3

**Published:** 2022-05-07

**Authors:** Themis Nikolas Efthimiou, Paul H. P. Hanel, Sebastian Korb

**Affiliations:** 1 Department of Psychology, University of Essex, Wivenhoe Park, Colchester, CO4 3SQ UK; 2 Department of Cognition, Emotion, and Methods in Psychology, University of Vienna, Vienna, Austria

**Keywords:** Facial neuromuscular electrical stimulation, User concerns, Risks, Burns, Pain

## Abstract

Facial neuromuscular electrical stimulation (NMES) is the application of an electrical current to the skin to induce muscle contractions and has enormous potential for basic research and clinical intervention in psychology and neuroscience. Because the technique remains largely unknown, and the prospect of receiving electricity to the face can be daunting, willingness to receive facial NMES is likely to be low and gender differences might exist in the amount of concern for the sensation of pain and skin burns. We investigated these questions in 182 healthy participants. The likelihood of taking part (LOTP) in a hypothetical facial NMES study was measured both before and after presenting a detailed vignette about facial NMES including its risks. Results showed that LOTP was generally high and that participants remained more likely to participate than not to, despite a decrease in LOTP after the detailed vignette. LOTP was significantly predicted by participants’ previous knowledge about electrical stimulation and their tendency not to worry about the sensations of pain, and it was inversely related to concerns for burns and loss of muscle control. Fear of pain was also inversely related to LOTP, but its effect was mediated by the other concerns. We conclude that willingness to receive facial NMES is generally high across individuals in the studied age range (18–45) and that it is particularly important to reassure participants about facial NMES safety regarding burns and loss of muscle control. The findings are relevant for scholars considering using facial NMES in the laboratory.

**Supplementary Information:**

The online version contains supplementary material available at 10.1186/s40359-022-00827-3.

## Introduction

Neuromuscular electrical stimulation (NMES) is a non-invasive technique to produce muscle contractions by delivering a current to the muscle through surface electrodes applied to the skin. This technique exploits the human body’s natural electrical characteristics (i.e., its’ conductivity and resistance) and manipulates them in a controlled way to induce motor action potentials. Stimulation intensity is typically applied at one of three thresholds: (1) the sensory threshold, at which people feel light tingling sensations; (2) the motor threshold, at which weak (visible) muscle contractions are produced; and (3) the functional threshold, at which a maximal muscle contraction is observed [1, 2]. NMES is mainly applied to the limb or trunk muscles and is popular for sports rehabilitation [3], and for restoring the function of paralysed muscles [4]. It is also used for brain research, as it allows for the investigation of both motor and sensory nerves [5–7].

NMES is considered a safe technique relative to other electrical stimulation techniques commonly used in psychological research (e.g., transcranial Altering and Direct Current Stimulation). Indeed, the possibility of inducing injuries with NMES is low, as long as the stimulation parameters are carefully selected [8], the device used complies with the International Electrotechnical Commission guidelines [9], and administrators are trained to follow safety guidelines [10]. Outside of the face, there has been only one reported case of burns as a result of deviation from the established protocol [11]. The most frequent NMES side-effects are pain and discomfort usually caused by inadequate electrode placement and high levels of impedance between the skin and electrode. In one study, 15% of participants who received NMES of the limbs described ‘prickling’ sensations [12]. Further, pain/discomfort was the stated reason for one in 68 [13] and one in 9 [14] participants to withdraw from an NMES study. Another concern is skin irritation (redness of the skin underneath the surface electrode), which is short-lived fading after 20–30 min [15]. Skin irritation can occur due to excessive ‘Joule heating’, namely the build-up of heat due to current resistance in the skin [16, 17].

To date, NMES has rarely been used in the face for scientific purposes, and even less so in healthy participants (although private companies claim that their commercial devices result in “facial toning” [15]. Some studies have applied NMES to the face in clinical populations, for example, to treat facial paralysis [18–20], symptoms of depression [21], and assist individuals with dysphasia with swallowing of food [22]. An additional three studies, have used facial NMES to explore afferent feedback to the central nervous system [23–25]. A final two studies have investigated if NMES-induced activation of specific facial muscles (e.g. the zygomaticus major involved in smiling) can alter the mood of healthy participants, and have found mixed results [26, 27]. The investigation of the effects of facial NMES in healthy participants clearly remains in its infancy.

There are two important challenges for the use of facial NMES research in psychology. One is the influence of participants’ anticipated concerns (e.g. of being in pain) on the phenomenon of interest, for example, their emotional experience [26]. In line with this, the literature on pain expectation [28] finds that the anticipation of pain impacts one’s subjective experience and neural processing of painful stimulation (for review see, [29]). When interviewing patients before they received an electrical stimulation garment for rehabilitation purposes, Moineau et al. [30] reported that participants were concerned about suffering burns and painful shocks but were nevertheless willing to use the garment for the benefits it offered. Consequently, if concerns are not adequately addressed participants’ experience with facial NMES may be modulated by their anticipated concerns, thereby confounding the results of the research.

A second obstacle is difficulty in recruiting and retaining participants, as they are likely to be concerned about potential side effects. Despite being a safe procedure when limits are followed and precautions are taken, the fact that NMES applies an electric current to the face contributes to making it appear dangerous and/or painful to the eyes of naïve volunteers. Volunteers may be deterred by the potential risks considering the negative consequences of damage to the body [31]. This concern is greater for facial NMES, because of the importance, the face has for nonverbal communication, and also because it quickly reveals an individual's sex, age, and attractiveness [32, 33]. Further, if skin irritation takes place on the limbs or trunk it can easily be concealed from view. In contrast, any burns or marks caused by NMES on the face are considerably more noticeable and can negatively affect one’s psychological well-being [34] and social interactions, as individuals with facial injury are perceived negatively and judged as less trustworthy and competent [35]. Nonetheless, the precise concerns that healthy individuals have about receiving NMES—particularly in the face—remain unknown.

### Potential predictors of the likelihood of taking part in a facial NMES experiment

How much these concerns about, and the willingness to receive, facial NMES vary based on demographic (e.g., gender, education, and age) and individual difference variables (e.g., personality, concerns), remains unknown to date. These factors seem relevant, as they have also been found to influence risky behaviour in general [36], and facial NMES might be considered risky by naïve individuals. For example, there is a clear gender disparity in risk-taking in choices on safety and health, with women being more risk-opposed than men [37, 38]. Additionally, as stated, one of the risks of NMES is that it may create skin irritation, which can impact one’s body image. Fear of facial skin irritation and burns is likely to be greater in women since they are generally more concerned with their body image than men [39, 40]. There exists also a distinction between men and women within their pain threshold and tolerance in experimental tasks [41, 42], which is modulated based on gender role beliefs [43], (for review see [44]). Gender and gender beliefs can also impact thresholds for expected pain tolerance during the anticipation of a fictitious electrical stimulation to the finger. For example, Pool et al. [45] found a substantial difference in reported pain tolerance between men and women who strongly identify with their gender role, compared to those who weakly identify with their gender role. Therefore, there may be gender differences in the overall willingness to participate in facial NMES research, as well as in the prevalence of specific concerns.

Additionally, gender-independent personality characteristics might also determine participants’ likelihood to take part in research involving facial NMES. Older populations tend to be more risk-averse than younger groups and may be less interested in participating in this type of research [46]. Similarly, participants may be more reluctant to receive facial NMES when they have particularly high or low levels of interoceptive awareness, which is known to play a role in the experience of pain [47, 48]. How people think of and perceive facial NMES could also differ by their motivation to approach or avoid emotion-inducing situations, as measured by the need for affect scale [49]. Indeed, research has shown that the need for affect is positively associated with risky behaviours, such as sensation-seeking and drug consumption [50]. Similarly, personality characteristics can also influence one’s motivation to engage in new and risky activities [51]. Two traits of the five-factor model, neuroticism and openness, seem particularly relevant. Individuals high in neuroticism also have high levels of anxiety, and report greater negative affect in their daily lives [52]. They might therefore be less inclined to participate in an NMES study, as they are more attentive to its risks and consequences [53]. Alternatively, neurotic individuals may be more inclined to participate in activities perceived as risky, due to their impulsivity and motivation to regulate their negative affect [51]. In contrast, individuals high in openness seek out new experiences that are abstract and intellectual [54, 55], and may therefore be more inclined to participate in psychological research using facial NMES.

## Method

### Study design and aims

The present online cross-sectional study was performed to better understand healthy people’s concerns about receiving facial NMES, and how these concerns differ by gender and personality characteristics. We made the following a priori hypotheses (pre-registered at https://osf.io/uf2ed/).

**H1**: Participants' concerns about skin burns, pain, and involuntary muscle movement are significant predictors of their willingness to take part in a hypothetical facial electrical stimulation study. Specifically, the higher the concern the less willing the subject is to take part.

**H2**: Participants' gender interacts with their concern about being burned, being in pain, and involuntary muscle movement. Specifically, concerns with pain and skin burns are, respectively, higher in men and women. This hypothesis was made on the assumption that female participants are more concerned about their physical appearance than men, especially in the face, and based on the popular belief that women tolerate pain better than men.

Moreover, we carried out exploratory analyses, e.g., to investigate if the need for affect is positively associated with the likelihood of taking part in a facial NMES study.

### Participants

A total of 233 people living in the UK, between the ages of 18 and 45, were recruited from the online platforms SONA (https://www.sona-systems.com; *n* = 68) and Prolific (https://www.prolific.co; *n* = 165). Participants were compensated financially or received course credits. We selected participants from those two pools to increase ecological validity: Researchers interested in recruiting participants for lab studies are likely to recruit from one of these pools. The research was approved by the ethics committee of the University of Essex (ETH2021-0744).

### Sample size justification

The sample size was estimated based on Schönbrodt and Perugini’s (2013) suggestion that a correlation with Rho = 0.2 and width = 0.15 stabilises with 197 participants. Therefore, we aimed to obtain data from slightly more than 200 participants, as some data loss was expected. Due to the novelty of the research effect sizes from previous work were unavailable.

### Measures

#### Likelihood of taking part

The main dependent variable was participants’ likelihood of taking part (LOTP) in a hypothetical facial NMES study. LOTP was measured at two-time points: at the beginning of the survey, after the hypothetical facial NMES research had been described with minimal information (LOTP1, see Additional file 1: S1), and later in the survey, after a comprehensive description of facial NMES and the associated risks had been provided (LOTP2 see Additional file 1: S2). At LOTP1 and LOTP2 participants answered the question ‘How likely are you to take part in a study involving facial neuromuscular electrical stimulation?’ using a 7-point scale, with the anchors 1 (*Extremely unlikely*), 4 (*Neither likely nor unlikely*), and 7 (*Extremely likely*). To ensure participants carefully considered their response, LOTP was measured one more time immediately after LOTP2. This LOTP3 rating (not pre-registered) used a slightly different wording: ‘How much do you intend to take part in a facial NMES study, if offered the possibility’. The 7-point Likert scale of LOTP3 had the anchors 1 (*I would never want to participate*)*,* 4 (*I am undecided about participating*)*,* and 7 (*I absolutely want to participate*). The two ratings of the likelihood of taking part after reading the detailed NMES descriptor (LOTP2 and LOTP3) produced nearly identical values (respectively, *M* = 4.84 and 4.88, *SD* = 1.77 and 1.75; α = 0.96) and were consequently averaged (henceforth called LOTP2). This was true in all but 19 participants, whose LOTP2 and LOTP3 values differed by more than two points, and who were therefore excluded from analyses (which however did not change the pattern of results, see Additional file 1: S5 Table).

Moreover, participants rated how much they agree with the statement that they feel concerned about (1) being burned, (2) being in pain, and (3) involuntary muscle movement, on a 7-point Likert scale with anchors 1 (*Strongly* disagree) to 7 (*Strongly agree*). These potential risks were included in the comprehensive description of facial NMES provided towards the end of the experiment (just before LOTP2 and LOTP3).

Additionally, we recorded through self-report participants’ gender, age, education, prior experience with electrical stimulation, theoretical and practical knowledge of electrical stimulation (0 *beginner* to 100 *expert*), and scores on five questionnaires.

#### Questionnaires

We measured approach and avoidance of emotions, using the Need for Affect Questionnaire (NAQ) [56], which measures motivation to approach emotion-inducing situations (e.g., “I feel the need to experience strong emotions”, 5-items, α = 0.85) and avoidance of emotion-inducing situations (e.g., “If I reflect on my past, I see that I tend to be more afraid of feeling emotions”, 5-items, α = 0.78). Risk-taking was measured using the Domain-Specific Risk-Taking Scale (DOSPERT) [57] specifically its subscale for health/safety (e.g., “Riding a motorcycle without a helmet”, 6-items, α = 0.55). To assess emotional distress or worry with sensations of pain or discomfort, we used the Not-Worrying subscale from the Multidimensional Assessment of Interoceptive Awareness (MAIA) [58], which includes five items (α = 0.80) such as: “I can stay calm and not worry when I have feelings of discomfort or pain”. The MAIA uses a 6-point Likert scale (*never*) 0–5 (*always*), but due to a programming error our version used 5-points but kept the same anchors (*never*) 1–5 (*always*). We reverse scored the MAIA subscale to make it more intuitive: high scores on the subscale reflect greater concern for or worry about sensations of pain or discomfort. Concerns with body image were measured using 20 items of the Body Image Concern Index (BICI) [59], which includes statements of the type “I try to camouflage certain flaws in my appearance”. Neuroticism (e.g., “I have frequent mood swings”, α = 0.71) and Intellect/imagination (e.g., “I have a vivid imagination”, α = 0.79) were assessed using the 4-item subscales taken from the mini International Personality Item Pool (mini IPIP) [60].

#### Procedure

The study was administered online using Qualtrics software (Provo, UT). Participants were told that we are interested in their opinions and beliefs regarding facial NMES and that they will complete questionnaires concerning risk, personality, body awareness, and body image.

**Fig. 1:**
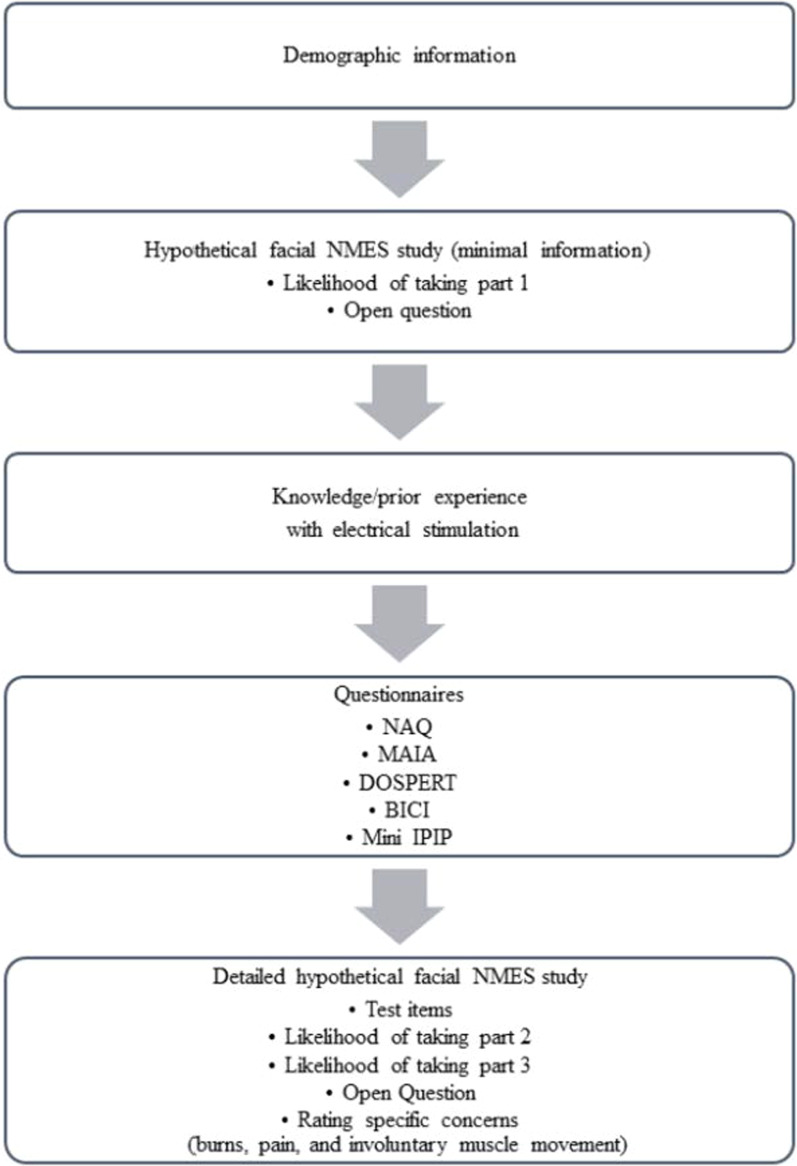
Flow diagram presenting the order of the survey administered to all participants

The order of measures is shown in Fig. 1 (full survey Additional file 1: S6). After providing consent and demographic information participants read a written description of a hypothetical scenario, in which NMES would be delivered to their face as part of a study in a psychological laboratory (Additional file 1: S1). Notably, the scenario described facial NMES as “a safe and non-painful technique” and provided little other information. Participants were asked to indicate on a 7-point Likert scale how likely they were to take part in the said hypothetical study (LOTP1) and subsequently could type through an open question what concerns they might have about taking part in the study. Thereafter, participants were asked about their knowledge of and experience with receiving electrical stimulation, and they then completed the five questionnaires. Subsequently, the hypothetical study was described in greater detail, highlighting the safety risks associated with facial NMES, such as burns, pain, and involuntary muscle movement, hereby called loss of muscle control. Some content questions were included, to ascertain that the descriptor had been read and understood. Participants were then asked again how likely they were to take part in the hypothetical study using two nearly identical questions (LOTP2 and LOTP3). Further, they were asked what concerns they might have—both using an open question and three separate Likert scales for ratings of the specific concerns of (1) pain, (2) burns, and (3) loss of muscle control.

#### Analyses

Data and analysis scripts are available online (https://osf.io/uf2ed/). The data were analysed following our pre-registered plan, after excluding participants who had given incorrect responses to two of the three content questions and test items, who had completed the survey in more than 15 min, or whose LOTP changed by two or more points in the two back-to-back items LOTP2 and LOTP3. Responses to LOTP1 and LOTP2 were analysed using separate step-wise multiple linear regressions using the *lm* function in R [61]. For the regression analysis, the continuous independent variables were cantered. Both models contained the initial predictors of: age, gender, level of education, knowledge of electrical stimulation (theoretical and practical), prior experience with electrical stimulation, as well as the five questionnaires. To reduce its terms, each model was run iteratively, progressively dropping terms with the largest *p* and smallest *t* value (backward elimination procedure). Then three moderated regressions were conducted to examine whether gender interacted with the specific concerns (burns, pain, and loss of muscle control). Finally, responses to open questions were assigned to one of ten themes, and summary statistics were computed. Additional exploratory analyses were also conducted. A third regression was conducted on the difference between LOTP1 and 2 to explore if any terms predicted a change in LOTP (LOTP2 minus LOTP1, larger values indicate a decrease in LOTP). Two one-sample *t*-tests examined whether LOTP1 and LOTP2 were above the neutral point of the scale (4: *neither likely nor unlikely*). In addition, three *t*-tests were used to compare men’s and women’s self-reported concerns for burns, pain, and Loss of muscle control. Finally, a mediation analysis was conducted on responses to LOTP2 using the package *Psych*.

## Results

The final analysis was conducted on a group of 182 participants between the ages 18–45 (*M*_*age*_ = 27.84, SD = 7.75), which is comprised of 90 men (*M*_*age*_ = 28.63, SD = 7.52) and 92 women (*M*_*age*_ = 27.07, SD = 7.93). Thirty-two people were excluded from analyses for failing one or more attention checks (filler questions included to determine data quality). An additional 19 participants were rejected, owing to the large change (two or more points out of seven) in their responses between two back-to-back items with slightly different wordings, measuring their likelihood of taking part (excluding those participants did not change the pattern of results, see analyses with 201 participants in Additional file 1: S5).

Overall, participants reported having low levels of theoretical and practical knowledge of electrical stimulation (see Table 2) (see Table 1).

**Table 1:** Summary of participants experience with some form of NMES using a purpose-built device

Experience with NMES	Count	%
No	128	25.27
Unsure	8	70.33
Yes	46	4.40
Sum	182	100
Reason		
Medical	19	41.30
Research	2	4.35
Other	25	54.35

In the following, we first describe the pre-registered analyses, central to our hypotheses (data and analysis script can be found online: https://osf.io/uf2ed/), followed by exploratory analyses).

### Pre-registered analyses

To examine the relations between all independent and dependent variables, a correlation matrix with Spearman correlations were produced (see Table 1). This showed that concern for pain was significantly inversely related to LOTP1 and that all three types of concerns (pain, burns, and loss of control) were significantly negatively correlated with LOTP2. Also notable, greater LOTP2 was found for participants who reported greater theoretical knowledge about electric stimulation, and who scored higher on the worrying subscale of the MAIA questionnaire.

To test our first hypothesis that concern for NMES-induced pain, burns, and loss of muscle control negatively predict the likelihood of taking part—, and to explore the contribution of individual differences as specified in the pre-registered exploratory analyses, two separate multiple regression analyses were conducted for LOTP1 and LOTP2.

### LOTP1: minimal knowledge of NMES

**Fig. 2:**
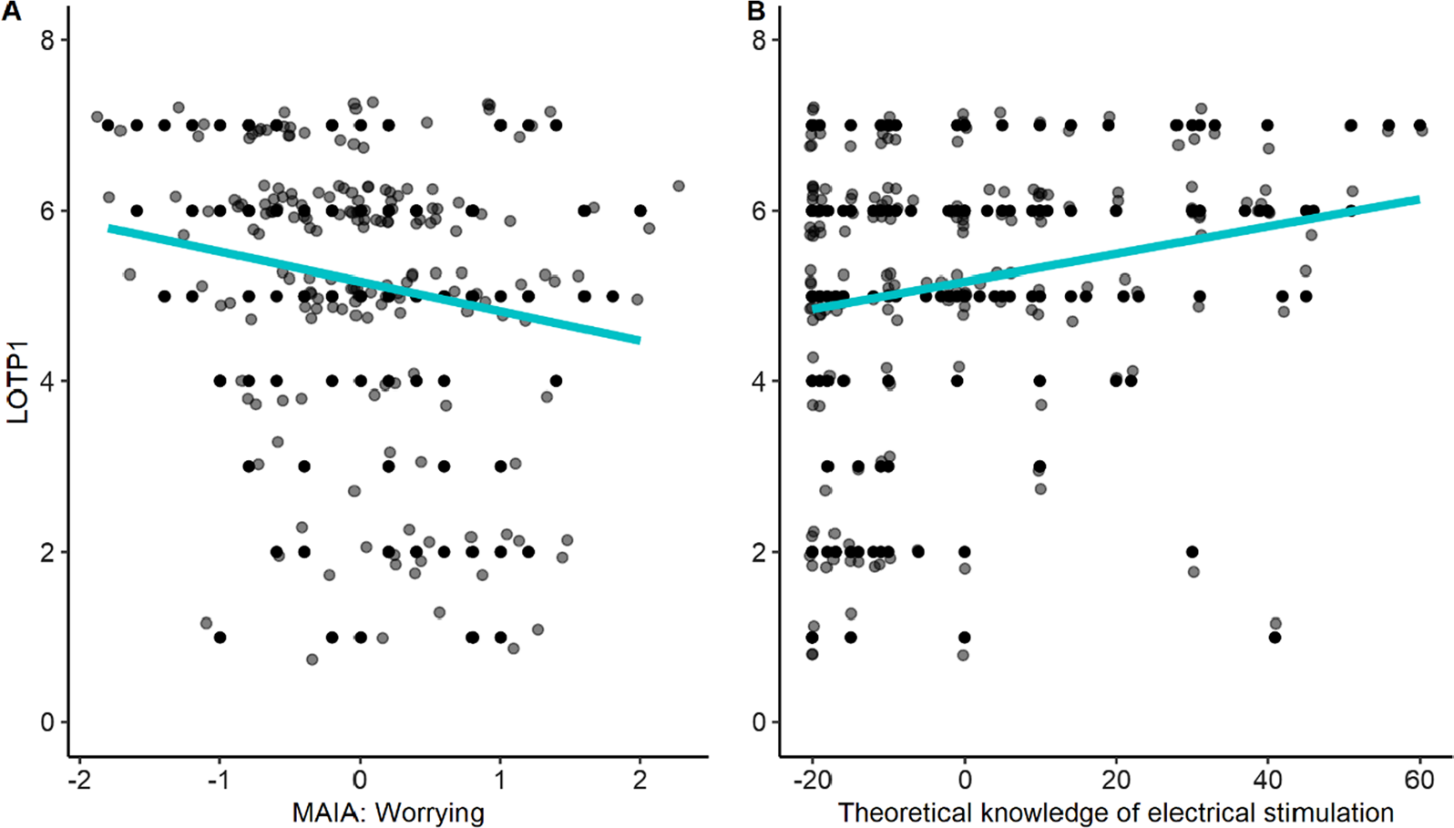
Scatterplots of significant terms predicting LOTP1. Participants’ LOTP1 increased significantly the less they worried about physical pain and discomfort, as measured with the (reversed) MAIA worrying subscale (**A**), and the more they knew about electrical stimulation (**B**). Model fit is shown by the blue line, black dots show individual data points (jittered in both dimensions to increase visibility)

Multicollinearity of the initial model was in the acceptable range, with all variance inflation factors below 10. The model was reduced until only two significant terms remained (Fig. 1): the level of prior theoretical knowledge of electrical stimulation (β = 0.02, *p* = 0.006), and MAIA’s worrying subscale (β =  − 0.35, *p* = 0.027). This reduced model explains a significant and small proportion of variance, *R*^2^ = 0.09, *F*(2, 179) = 8.32, *p* < 0.001; *adj. R*^2^ = 0.07 (Fig. 2).

### LOTP2: detailed knowledge of NMES

The initial model for LOTP2 included the same terms as for the initial model fitted on LOTP1, with the addition of scores for the three concerns, i.e., burns, pain, and loss of muscle control. Multicollinearity was in the acceptable range, with all variance inflation factors below 10.

**Fig. 3:**
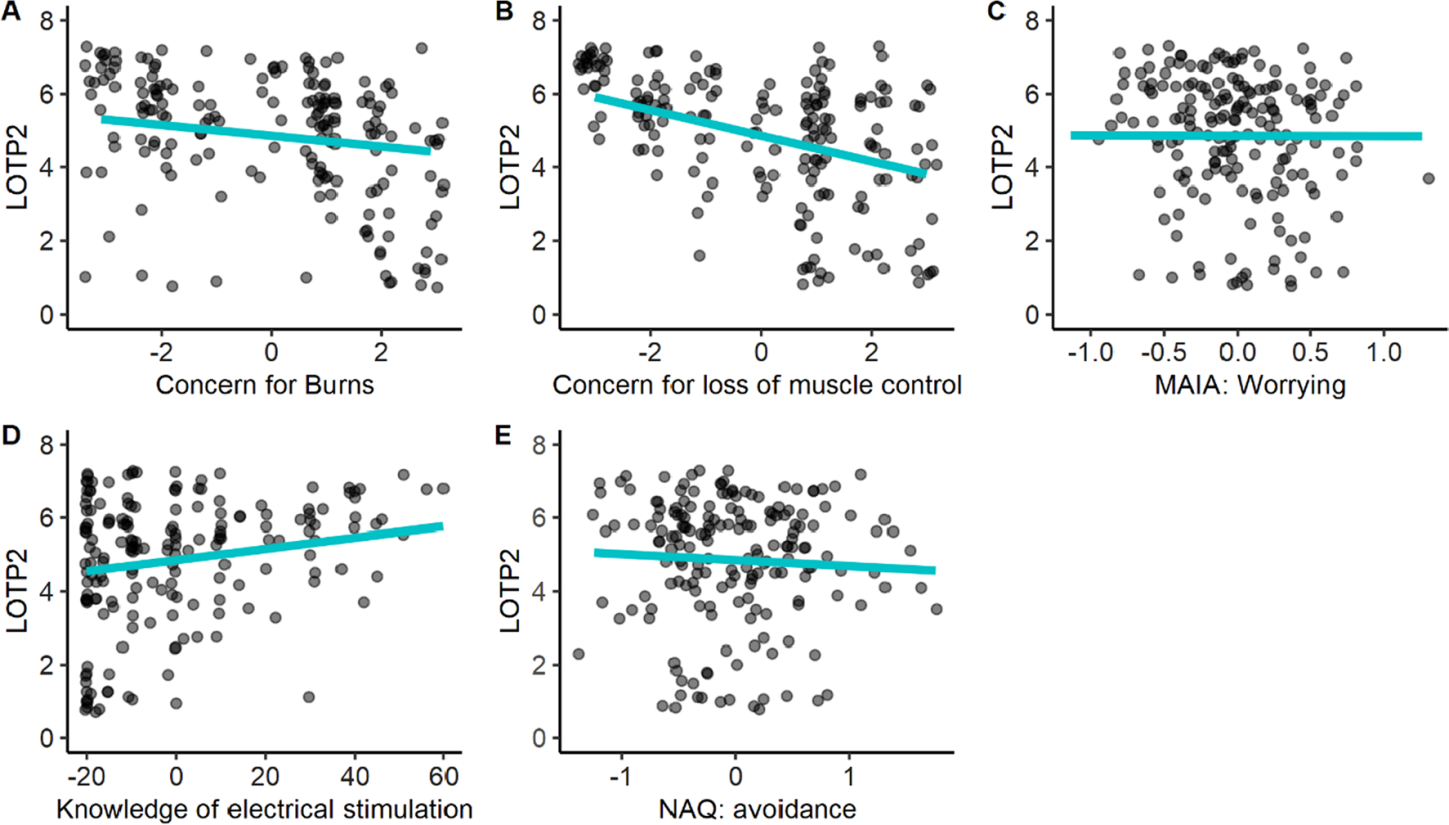
Scatterplots of significant terms predicting LOTP2. Likelihood of taking part in the NMES experiment significantly decreased the greater participants’ concern for burns (**A**) and loss of muscle control (**B**). Moreover, it increased significantly the less participants worried about physical pain and discomfort (**C**), the more they knew about electrical stimulation (**D**), and the higher their score on the NAQ avoidance (**E**). Model fits shown by the green line, individual data points by black dots (jittered to increase visibility)

The reduced model explains a significant and substantial proportion of variance, *R*^2^ = 0.37, *F*(5, 176) = 20.33, *p* < 0.001, *adj. R*^2^ = 0.35. The final model included three significant negative terms: concern for burns (β =  − 0.14, *p* = 0.022; Fig. 3A), concern for loss of muscle control (β =  − 0.30, *p* < 0.001; Fig. 3B), and the MAIA’s worrying subscale (β =  − 0.40, *p* = 0.011; Fig. 3C). In addition, there were two significant positive terms: theoretical knowledge of electrical stimulation (β = 0.01, *p* = 0.006; Fig. 3D) and surprisingly also the NAQ avoidance subscale (β = 0.21, *p* = 0.009; Fig. 3E).

To test our second hypothesis—of a significant interaction between the participants' gender and their concerns about being burned, being in pain, and losing muscle control—we ran three moderated regression analyses, one per concern, with the dependent variable being LOTP2 and the predictor gender and the concern. Only the model (full model descriptions in Additional file 1: S3 and Additional file 1: S4) for loss of muscle control produced a main effect (β =  − 0.49, *p* = 0.011), no other main effects were found (all *ps* > 0.059). Further, contrary to predictions, no significant interaction with gender was found in any of the models (all *p*s > 0.313).

To further capture participants’ thoughts about NMES, the open questions were reviewed and coded according to their content. A total of 10 categories emerged (see Table 2). Approximately 25% of participants reported having no concern or non-NMES related concerns, such as about the compensation or practicalities of travelling to a laboratory.

**Table 2:** Descriptive statistics and correlations for the likelihood of taking part before (LOTP1) and after (LOTP2) reading a detailed description of NMES and the study variables

Variables	M	SD	1	2	3	4	5	6	7	8	9	10	11	12	13	14
1. LOTP1	5.17	1.59	1.00***													
2. LOTP2	4.87	1.73	.56***	1.00***												
3. Theoretical know	20.05	20.25	.24	.28*	1.00***											
4. Practical know	11.21	17.19	.15	.21	.59***	1.00***										
5. NAQ-Approach	1.01	.93	− .02	− .03	.02	.07	1.00***									
6. NAQ-Avoidance	− .42	1.34	− .05	.06	− .15	− .14	− .30**	1.00***								
7. MAIA	3.00	.75	− .21	− .39***	− .23	− .27*	.16	.15	1.00***							
8. DOSPERT	18.83	6.26	.01	.18	.02	.01	− .14	.11	− .17	1.00***						
9. BICI	45.29	15.85	− .04	.05	− .06	− .07	.04	.42***	.16	.24	1.00***					
10. Neuroticism	11.66	3.45	− .10	− .09	− .19	− .14	.18	.46***	.43***	− .06	.47***	1.00***				
11. Openness	14.70	3.43	.08	.15	.19	.06	.09	− .02	− .12	.07	.01	.03	1.00***			
12. Burns	4.09	1.97	− .19	− .39***	− .11	− .14	.05	.09	.27*	− .03	.13	.10	− .14	1.00***		
13. Pain	4.14	1.87	− .29**	− .37***	− .16	− .14	.15	.01	.44***	− .04	.22	.15	− .19	.58***	1.00***	
14. Loss of muscle control	4.02	1.92	− .30**	− .52***	− .21	− .24	.16	.11	.45***	− .12	.13	.27*	− .09	.53***	.51***	1.00***

N = 182 for all correlations

Spearman correlation rho values. Statistical (bold) significance (corrected for multiple inferences with Holm’s method) is indicated by asterisks: * *p* < .05; ** *p* < .01; ***p < .001

NAQ, need for affect questionnaire; MAIA, multidimensional assessment of interoceptive awareness; DOSPERT, domain-specific risk-taking scale; BICI, body image concern index; loss of muscle control

### Exploratory analyses

As can be seen in Table 1, the average LOTP decreased after the detailed NMES description was presented. This reduction in LOTP was noted in 96 participants, whilst LOTP increased in 41 participants, and did not change in 45 participants. At LOTP1, 78.6% of the sample were ‘slightly likely’ or more to take part, and 21.4% were ‘unlikely’ or ‘unsure’. At LOTP2, 70.3% were ‘slightly likely’ or more to take part, and 29.7 were ‘unlikely’ or ‘unsure’. Therefore, we explored this decrease in LOTP, and whether it differed by gender, by fitting a linear model to predict LOTP, with the time of LOTP (1 and 2) were measured and gender (male and female) as predictors. The model explains a significant and very small proportion of variance, *R*^2^ = 0.02, *F*(3, 360) = 2.32, *p* = 0.075, *adj. R*^2^ = 0.01, but there were no significant main or interaction effects (all *ps* > 0.058). However, within the model the term gender was marginally significant (β =  − 0.33, *p* = 0.058), with overall LOTP being lower in female participants (*M* = 4.85, *SD* = 1.47) compared to males (*M* = 5.18, *SD* = 1.45).

To test whether LOTP1 and LOTP2 substantially differed from the neutral scale mid-point, we carried out two one-sampled *t*-tests testing the difference between the two LOTPs and μ = 4 (corresponding to the midpoint on the 7-point rating scale, labelled ‘*neither likely nor unlikely*’). The *t*-test for LOTP1 (*M* = 5.17) resulted in a statistically significant, medium-to-large effect size (difference = 1.17, 95% CI [4.94, 5.40], *t*(181) = 9.95, *p* < 0.001; *d* = 0.74, 95% CI [0.57, 0.90]). The *t*-test for LOTP2 (*M* = 4.86) resulted in a significant medium-sized effect (difference = 0.86, 95% CI [4.60, 5.11], *t*(181) = 6.70, *p* < 0.001; *d* = 0.50, 95% CI [0.34, 0.65]). Thus, in both cases participants were significantly more likely to take part than not to.

**Fig. 4:**
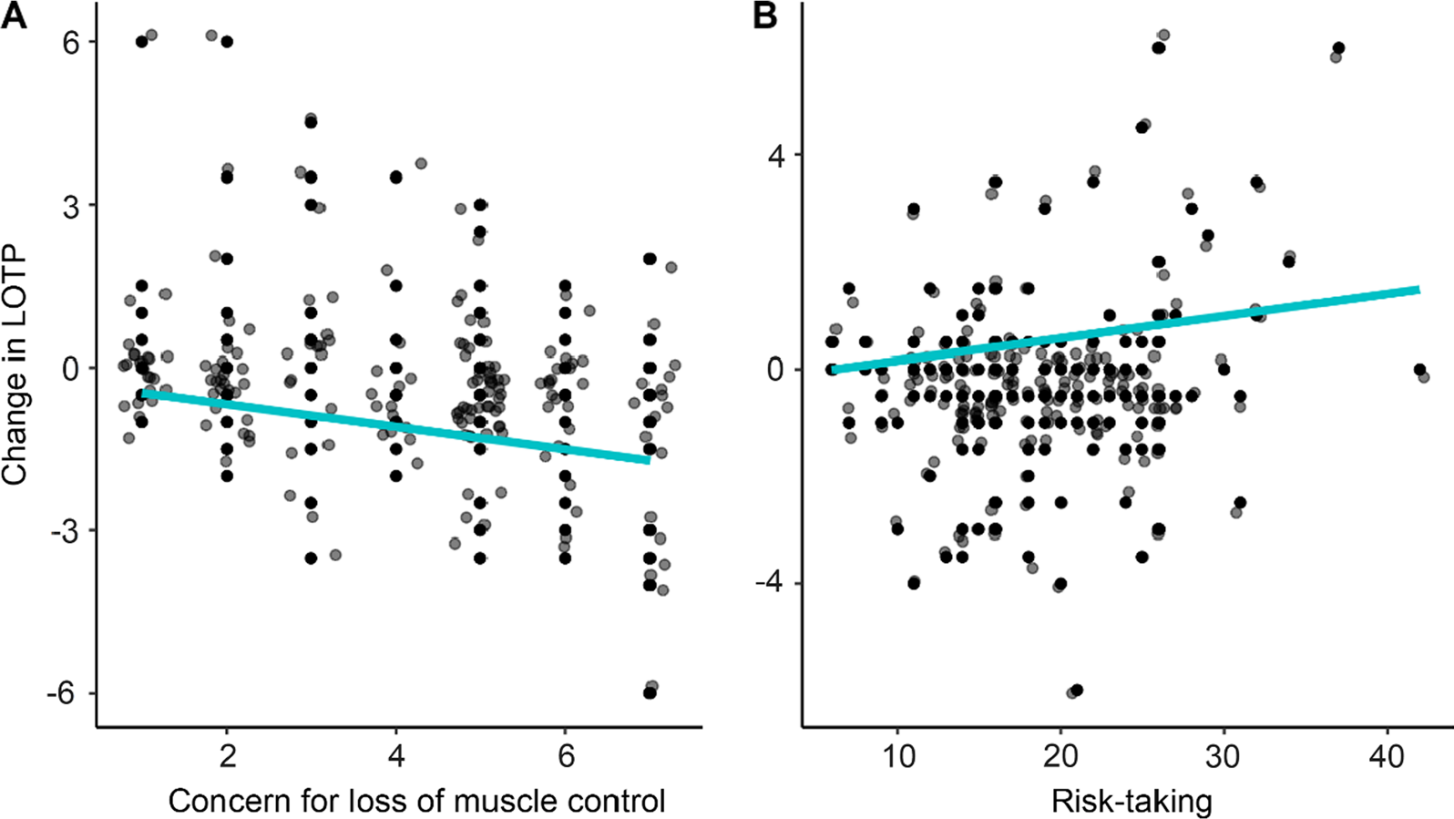
Scatterplots of significant terms predicting a change in LOTP after the detailed vignette was presented. The reduction in LOTP was (**A**) negatively predicted by concern for the loss of muscle control, and (**B**) positively predicted by risk-taking. Model fits are shown by the blue line

Next, we ran another model to explore further which variables explain the observed slight decrease in LOTP. To achieve this, we computed the change in LOTP (LOTP2 minus LOTP1) and fitted a multiple regression analysis with all terms previously used for the analysis of LOTP2. The model was reduced using the backward-elimination method until only significant terms remained: risk-taking (β = 0.25, *p* = 0.021) and concern for loss of muscle control (β =  − 0.21, *p* < 0.001). The model explains a significant and moderate proportion of variance, *R*^2^ = 0.10, *F*(2, 179) = 10.37, *p* < 0.001, *adj. R*^2^ = 0.09. Participants’ willingness to participate in the fictitious facial NMES study decreased after they received more detailed information about the associated risks, and this decrease was larger (more negative values) the greater the concern for losing control of facial muscles (Fig. 4A), and it was smaller (more positive values) the higher the risk-taking score (Fig. 4B).

Next, we tested if our sample shows a significant difference in risk-taking by gender, as reported in the literature [38]. A two-sample *t*-test on the health and safety subscale of the DOSPERT found a non-significant marginal difference between male and female participants, *t*(180) = 1.89, *p* = 0.065. As expected, female participants had lower risk-taking scores (*M* = 17.97, *SD* = 1.87) than male participants (*M* = 19.71, *SD* = 1.77).

As participants’ gender did not predict LOTP1 or LOTP2 and did not interact with specific concerns, we explored whether ratings for concerns differed between men and women. Using two-sample *t*-tests, we found greater concern in female than male participants across all three types of concerns (Table 3).

**Table 3:** The number of participants indicating concerns in open questions, as well as their average (SD) LOTP, before and after reading a detailed description of facial NMES and its risk

Categories of concern	Before			After		
	*n*	LOTP1 (M)	LOTP1 (SD)	n	LOTP2 (M)	LOTP2 (SD)
No or non-NMES related concerns	49	5.55	1.50	63	5.28	1.69
Skin burns and irritation	4	5.75	2.40	31	4.68	1.65
Pain and discomfort	39	4.92	1.99	30	5.17	1.47
Pain and burns/irritation	4	5.50	1.11	11	3.27	1.74
Involuntary muscle movement and appearing odd	7	5.57	.95	9	4.56	2.26
Immediate or long-term damage to the face/nerve	40	5.45	1.30	12	4.92	1.14
Lack of information and unfamiliarity with the sensation or technique	25	4.36	1.80	8	4.81	1.81
Interaction with a pre-existing health condition	0	0	0	3	2.83	1.61
Concerned but no specific reason	11	4.73	1.86	11	4.41	1.81
Faulty machine or lack of trust in administrator	3	4.67	3.33	4	5.00	2.12

**Fig. 5:**
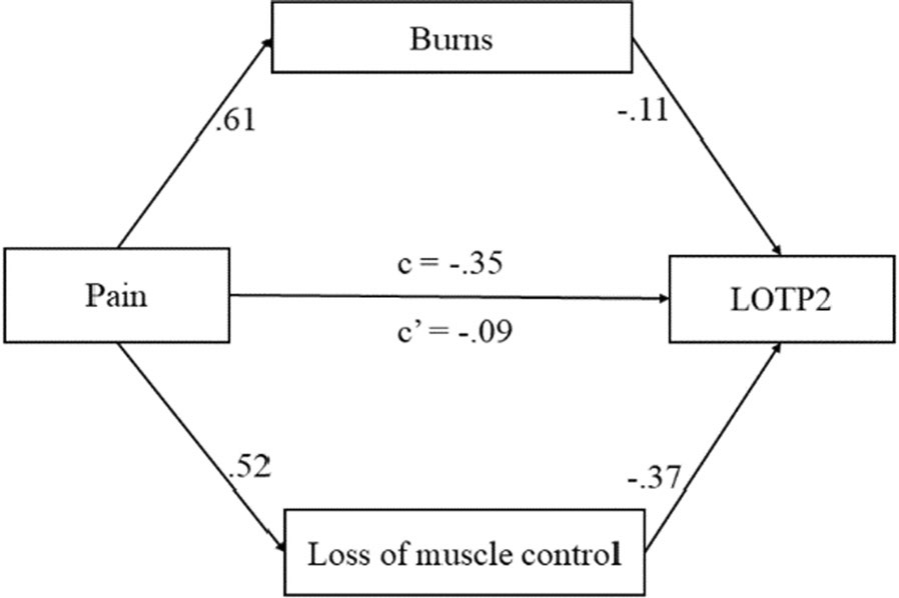
Regression coefficients for the relationship between the participant’s concern for Pain and LOTP2 as mediated by concern for burns and loss of muscle control

Furthermore, we conducted a mediation analysis to explore the relationship between the three concerns and their impact on LOTP2 (Fig. 5). The effect of concern for pain on LOTP2 was fully mediated via the concern for burns and loss of muscle control.

The regression coefficient between concern for burns and LOTP2 and the regression coefficient between concern for loss of muscle control and LOTP2 were both significant. The total effect (c) of pain on LOTP2 is − 0.35, *SE* = 0.06, *t*(180) = 5.40 with *p* =  < 0.001. The direct effect (c′) of concern for pain on LOTP2 removing the concern for burns and loss of muscle control is − 0.09, *SE* = 0.07, *t*(178) = 1.17 with *p* < 0.001. The mean bootstrapped indirect effect of pain on LOTP2 through the concern for burns and loss of muscle control is − 0.26 with *SE* = 0.05, 95% CI [− 0.36, − 0.16], *R* = 0.55, *R*^2^ = 0.30, *F*(3, 178) = 25.08, *p* =  < 0.001.

A final analysis compared this study’s sample characteristics on the four questionnaires to the samples from previous research, using two-sample tests. The current sample’s mini IPIP score (including the subscales Neuroticism and Intellect/Imagination) was compared to a confirmatory analysis of the mini IPIP [62]. For NAQ [56], DOSPERT [57], and BICI [59] we compared our sample to the original samples used in the development of those questionnaires. Compared to the literature, our participants were found to score significantly lower (see Table 4) in risk-taking (measured with the health and safety subscale of DOSPERT), intellect/imagination (subscale of IPIP), and dysmorphic concern (BICI) (Table 5).

**Table 4:** Mean differences in self-reported concern for pain, burns, and loss of muscle control between male and female participants

*Concern*	*t*	*p*	Mdiff 95% CI	*Cohens d*	*M*_*male*_	*M*_*female*_
Pain	3.20	.002	[.33, 1.40]	.48	3.70	4.57
Burns	3.61	< .001	[.46, 1.58]	.54	3.58	4.60
Loss of muscle control	3.37	< .001	[.39, 1.48]	.50	3.54	4.48

All two-sample *t*-tests had 180 degrees of freedom

**Table 5:** Mean differences between our sample and those reported in prior research across four questionnaires utilised in this study

Questionnaire	Subscale	*t*	*df*	*d*	*p*	*M*_*prior*_	*SD*_*prior*_	*M*_*current*_	*SD*_*current*_
IPIP	Neuroticism	.52	1662	.04	.303	11.81	3.72	11.66	3.49
	Intellect/Imagination	4.49	1662	.35	< .001	15.81	3.11	14.70	3.44
NAQ	Avoidance	1.04	416	.10	.149	− .55	1.20	− .42	1.34
	Approach	.10	416	.01	.458	1.02	1.00	1.01	.93
DOSPERT	Health/Safety	2.51	352	.27	.006	20.63	7.43	18.83	6.26
BICI		3.25	364	.34	.001	50.40	14.20	45.29	15.85

The column M and SD prior contain mean and standard deviation reported in the prior literature this studies sample is being compared to, which in turn is reported as M and SD current. We did not include the MAIA worrying subscale, as we scored it differently to the literature

## Discussion

The present pre-registered cross-sectional study investigated the concerns of individuals about facial NMES, and their likelihood of taking part (LOTP) in a hypothetical study that uses facial NMES. Moreover, it explored whether LOTP changes depending on the level of information provided about facial NMES and its risks (comparing LOTP across two-time points) and whether LOTP differed depending on demographics and individual differences.

The results are in line with our first hypothesis (H1), which stated that LOTP is lower among participants who are more concerned about the risks described to them, that is the risks of burns, pain, and loss of muscle control. Indeed, we found that all three concerns were significantly negatively correlated with LOTP1 and LOTP2 (Table 1). This finding is unsurprising and consistent with theories of decision-making, which propose that the risks/costs of an action are weighed against its benefits [63, 64]. However, only concerns for burns and loss of muscle control emerged as significant predictors of LOTP2, such that greater concern resulted in a lower likelihood of taking part. As indicated by a mediation analysis, concern for pain was mediated by the other concerns (Fig. 5). Thus, while pain is a real concern, burns and loss of muscle control appear to be more important for participants’ decision-making process. Therefore, when recruiting participants for an experiment using facial NMES, and upon arrival to the lab, researchers should prioritise addressing potential concerns about burns and loss of muscle control. For example, to address concerns of loss of muscle control, it could be emphasised that participants can remove the electrodes at any time (or hit a “stop” button) if they feel they are losing control over their facial muscles.

It is also noteworthy that in the first open question (prior to the mention of facial NMES related risks), participants reported being concerned for the three concerns we later highlighted, with pain being the most commonly evoked. Nevertheless, the number of times participants mentioned feeling concerned about skin burns and markings increased between the beginning and the end of the experiment, that is after receiving more detailed information about NMES. Surprisingly, nerve damage or facial paralysis was the second most common risk participants believed to be associated with facial NMES, and our data suggest that this concern can be reduced by providing a more detailed description of facial NMES (its prevalence dropped from 40 to 12, see Table 2). We suggest that to reduce their negative effect, volunteers’ self-reported concerns should be acknowledged by researchers and that they should be directly addressed during recruitment and upon arrival to the lab.

Additional unregistered analyses were carried out to explore the influence of informing participants about the risks associated with facial NMES on their overall LOTP. We found that providing detailed information about facial NMES and the possible risks slightly reduced LOTP, indicating that participants were less likely to take part at time point two (*M* = 4.86, *SD* = 1.73) compared to time point one (*M* = 5.17, *SD* = 1.59)—yet this difference was not statistically significant, and participants remained more likely to take part than not to.

Finally, we looked at which variables explain a change in LOTP after a detailed description of NMES was given (LOTP2 minus LOTP1). The concern for the loss of muscle control predicted a decrease in LOTP, thus it should be one of the main concerns addressed by research interested in using facial NMES. This finding is consequential for research interested in applying NMES above the sensory threshold and may be reduced by applying low-intensity NMES to the limb before the face to introduce the sensation and technique. Lastly, we find that high-risk takers’ LOTP increased after the detailed NMES description. These results are relevant to psychological research as the risks of participating in a facial NMES must be stated to acquire informed consent. Therefore, researchers should address the specific concerns, possibly by explaining how burns occur and what safety measures have been put in place to minimise them.

To test our second hypothesis, (H2), we examined whether concerns about burns, pain, and loss of muscle control interacted with gender in predicting LOTP. Overall, men and women are equally likely to take part in facial NMES studies. Interestingly, we have found that women and men differed in their concerns (see Table 3). Men were less worried by all three risks compared to women, contrary to our hypothesis that men will be more concerned with pain. Crucially, this did not influence LOTP. Speculatively, this may be due to gender norms, with male participants trying to appear more stoic and less concerned about pain [42, 45]. The finding of lower concern for pain in male participants is also in line with reports of greater tolerance for both actual and expected pain in male participants, especially in those conforming to traditional gender roles [41, 42, 45].

Another possibility is that the greater concern for pain, burns, and loss of muscle control in females than male participants stems from differences in risk-taking between men and women in the domain of health and safety [65]. Indeed, although the difference was only marginally significant (*p* = 0.065), we did find lower risk-taking scores in female than male participants. It should also be noted that our sample consists of overall low-risk takers, with both genders reporting to be on average ‘*unlikely*’ to engage in risky activities. This general risk aversiveness could be due to the study being carried out during the covid19 pandemic (January 2021), although only one participant mentioned concerns relating to covid19 when answering the open questions. In summary, LOTP did not differ significantly by demographic characteristics, which suggests that these factors can be (mostly) disregarded when designing recruitment materials or information sheets for facial NMES research.

To further explore the effect of demographic and individual differences on LOTP, we conducted exploratory analyses including the questionnaires. For both LOTP1 and LOTP2, the ‘worrying subscale’ of the MAIA was found to be a significant negative predictor (Figs. 2A, 3D). Specifically, the more participants tended to be worried or experience emotional distress with sensations of pain or discomfort, the less likely they were to take part in the hypothetical facial NMES study. It might be possible to reduce participants’ worries about pain and discomfort by making them more mindful [66]. However, it does not seem practical to always include a mindfulness intervention in a laboratory study on facial NMES. Therefore, researchers should instead assess potential participants’ a priori propensity for suffering pain, as participants who are less concerned with uncomfortable physical sensations may better tolerate the effects of facial NMES.

Another significant predictor of LOTP, both at times one and two, was found to be one’s self-reported theoretical knowledge of electrical stimulation (Figs. 2B, 3E). Interestingly, this result seems at odds with our other findings that LOTP decreases when participants are given more detailed information about facial NMES. Although the information given to the participants was mainly related to risks, future research should aim to examine in more detail how the initial description of facial NMES influences volunteers’ willingness to participate in the study, depending on their prior knowledge about electrical stimulation.

Surprisingly, the tendency to avoid emotions and emotion-inducing situations, measured with NAQ’s avoidance subscale, predicted greater LOTP2 (Fig. 3C). The finding was unexpected and seems counterintuitive. However, it is unlikely to originate from an anomaly in our sample, as NAQ avoidance scores were similar to those reported in previous research [56] (see Table 4), and were positively correlated with neuroticism (*r* = 0.46, *p* < 0.001, see Table 2)—as expected based on the literature [49]. Speculatively, the positive link between NAQ avoidance and LOTP2 is due to the facial NMES descriptor. Prior research suggests that individuals with a high NAQ-approach are more attentive and immersed in emotional narratives [67]. For these subjects, facial NMES may not have appeared as an emotion-inducing event.

The description provided in the current study mimics the typical process of recruiting participants for laboratory-based studies, by presenting an advert describing the study’s aims, as well as details about compensation and risks involved. Our findings demonstrate that the type and level of information presented influences participants’ decision to take part in a laboratory study involving facial NMES. Providing more information about the potential risks linked to facial NMES tends to reduce participants likelihood of taking part. Importantly, however, the majority of participants remained willing to take part in the hypothetical study even after reading such information. The current study cannot inform us about how much the information provided influences participants’ inclination to withdraw from such a study once they already accepted to take part. To answer that question, the variables influencing the likelihood to take part, and the likelihood not to withdraw prematurely, need to be explored in a laboratory setting. In an actual laboratory experiment, participants can typically ask questions and interact otherwise with the experimenter(s). Therefore, it is of paramount importance that experimenters establish a relationship of trust with participants. Overall, for psychological research using facial NMES, it is important to gain insight into the concerns that participants might have. This aspect seems particularly relevant when examining the effects of proprioceptive feedback on mood [26], as participants’ concerns may induce negative affect, which in turn could confound the experimental results.

There are several limitations to considered. First, as there is limited research in this area, therefore the sample size is based on suggestions for the statistical analysis, rather than prior work or power analysis based on existing effect sizes. Therefore, the power of the statistical analyses is unclear. Second, we did not exclude participants who may be unable to partake in research using facial NMES, such as individuals with pacemakers, the responses may be largely influenced by this concern than the one’s of interest. However, as only three participants reported concern for facial NMES interacting with a pre-existing condition, this would have not skewed the results. Third, the outcome measure use for our statistical analysis "Likelihood of taking part" is a novel measure that has yet to be validated. Although, research has used single-item questions as a method to capture participants self-perception [45, 68]. Last, participants responded to a hypothetical scenario, and it is unclear how well it generalises to real world scenarios. Although, in the context of pain induced by electrical stimulation Pool et al. [45] suggests participants reports to a hypothetical scenario resemble actual behaviour in the lab.

In summary, healthy participants aged 18–45 are generally likely to take part in facial NMES research, and this remains true even after highlighting to them the risks associated with facial NNES. Furthermore, the most important concerns standing in the way of participants’ participation in such research relate to skin burns and involuntary muscle movement (loss of muscle control). Fear of pain is also a major concern but seems mediated by the other two points. Finally, the choice to participate in a laboratory study involving the administration of facial NMES does not seem to differ by gender, age, and education level; instead, it depends on people’s prior knowledge about electrical stimulation, and their propensity to worry about sensations of pain or discomfort.

## Supplementary Information

**Additional file 1**. Supplemental Materials.

## Data Availability

The dataset supporting the conclusions of this article along with the materials and scripts are available on the Open Science Framework (OSF): https://osf.io/uf2ed/. The experiment was preregistered.

## Abbreviations

NMES: Neuromuscular Electrical Stimulation
MAIA: Multidimensional Assessment of Interoceptive Awareness
LOTP: Likelihood of taking part
IPIP: International Personality Item Pool
NAQ: Need for Affect Questionnaire
DOSPERT: Domain-Specific Risk-Taking
BICI: Body Image Concern Index
