# Supplementary material for: Volunteers’ concerns about facial neuromuscular electrical stimulation
Source: BMC Psychol. 2022 May 7;10:117. doi: 10.1186/s40359-022-00827-3 (PMC9080168; doi:10.1186/s40359-022-00827-3)
Supplement: Supplementary file 1 — Additional file 1. Supplemental Materials. [file 40359_2022_827_MOESM1_ESM.docx]

**Volunteers’ concerns about facial neuromuscular electrical stimulation**

Supplemental Materials

**S1.**

**The first hypothetical study presented with minimal information about facial NMES.**

“*Imagine the following situation: You are invited to a research laboratory to take part in a paid (£10 per hour) psychological experiment about emotions. This study will use facial neuromuscular electrical stimulation, a safe and non-painful technique, to stimulate certain parts of your face with weak electrical impulses.*”

**S2.**

**Hypothetical study 2 with high levels of information about facial NMES and its’ risks.**

“*PLEASE READ CAREFULLY, as you will have to answer questions based on the text below. We will now provide you with a more detailed description of what NMES is, and of how we plan to use it in a hypothetical, planned research experiment. What is NMES: Neuromuscular Electric Stimulation (NMES), also known as TENS (Transcutaneous Electrical Nerve Stimulation), has been used for some time, especially in the field of physiotherapy and rehabilitation, including in the face. NMES can be used to induce muscle contractions, as it simulates the natural activation of a muscle. How do we intend to use NMES: For the purpose of a planned experiment, four electrodes will be placed on your lower face. These are medically suitable, self-adhesive electrodes, which are disposed after each use. They are used for light electrical muscle stimulation, which takes place with medically certified and computer-controlled stimulators. You will receive NMES over specific facial muscles, mostly in short periods of approximately 1 second, and for a maximum total duration of 30 minutes. You will be compensated with £10 per hour. You are allowed to stop participating at any time. Side effects and Risks: Discomfort, Pain: Most of the time, facial NMES will feel like a slight, non-painful tingling sensation. However, it can sometime feel unpleasant, and occasionally induce brief painful sensations. The level of pain/discomfort depends on many factors, including individual differences in pain sensitivity. Marks, burns: The risk of getting skin burns exists, but is very low, as long as safety measures are followed, and stimulation limits are not exceeded. In some cases, however, light skin redness due to irritation can occur under the electrodes. This should disappear after few hours. Loss of control: Be aware that your muscles will move without you controlling it. Your facial muscles may also feel tight and tired at the end of the experiment. Although not a health risk, these phenomena can be unpleasant for some people. Other: For people with heart problems, a pacemaker or other implantable heart device, NMES may be dangerous and is not recommended. It is also not recommended during pregnancy. PLEASE READ CAREFULLY, as you will have to answer questions based on the text above.*”

**S3.**

Table of full model results from moderated linear mixed effects regressions examining whether specific concern (pain, burns, and LoC) predict LOTP2 from 182 participants. F-values are from ANOVAs with Satterthwaite method degrees of freedom.

| Model | Term | Result |
| --- | --- | --- |
| Gender *Burns | - | *R*^2^ = .16, *F*(3, 178) = 11.27, *p* < .001, adj. *R*^2^ = .15 |
|  | Gender | β = -.03, 95% CI [-.51, 0.45], *t*(178) = -.12, *p* = .905 |
|  | Burns | β = -.16, 95% CI [-.54, 0.23], *t*(178) = -.80, *p* = .425 |
|  | Burns – Gender | β = .13, 95% CI [-.37, 0.12], *t*(178) = 1.01, *p* = .313 |
| Gender * Pain | - | *R*^2^ = .14, *F*(3, 178) = 9.70, *p* < .001, adj. *R*^2^ = .13 |
|  | Gender | β = .09, 95% CI [-.57, .40], *t*(178) = -.35, *p* = .726 |
|  | Pain | β = -.41, 95% CI [-.83, -.02], *t*(178) = -1.90, *p* = .059 |
|  | Gender - Pain | β = -.04, 95% CI [-.22, .30], *t(*178) = -.33, *p* = .744 |
| Gender * Loss of muscle control | - | *R*^2^ = .27, *F*(3, 178) = 22.26, *p* < .001, adj. *R*^2^ = .26 |
|  | Gender | β = -.06, 95% CI [-.39, .51], *t*(178) = .27, *p* = .790 |
|  | Loss of muscle control | β = -.49, 95% CI [-.86, -.12], *t*(178) = -2.58, *p* = .011 |
|  | Gender - Loss of muscle control | β = -.01, 95% CI [-.22, .24], *t*(178) = -.08, *p* = .937 |

**S4.**

Plots of the moderated regression results examining whether specific concerns (Burns, Pain, and loss of muscle control) interacted with the participant’s gender to predict LOTP2. Model fit is shown by the red line, shading shows 95% confidence interval.


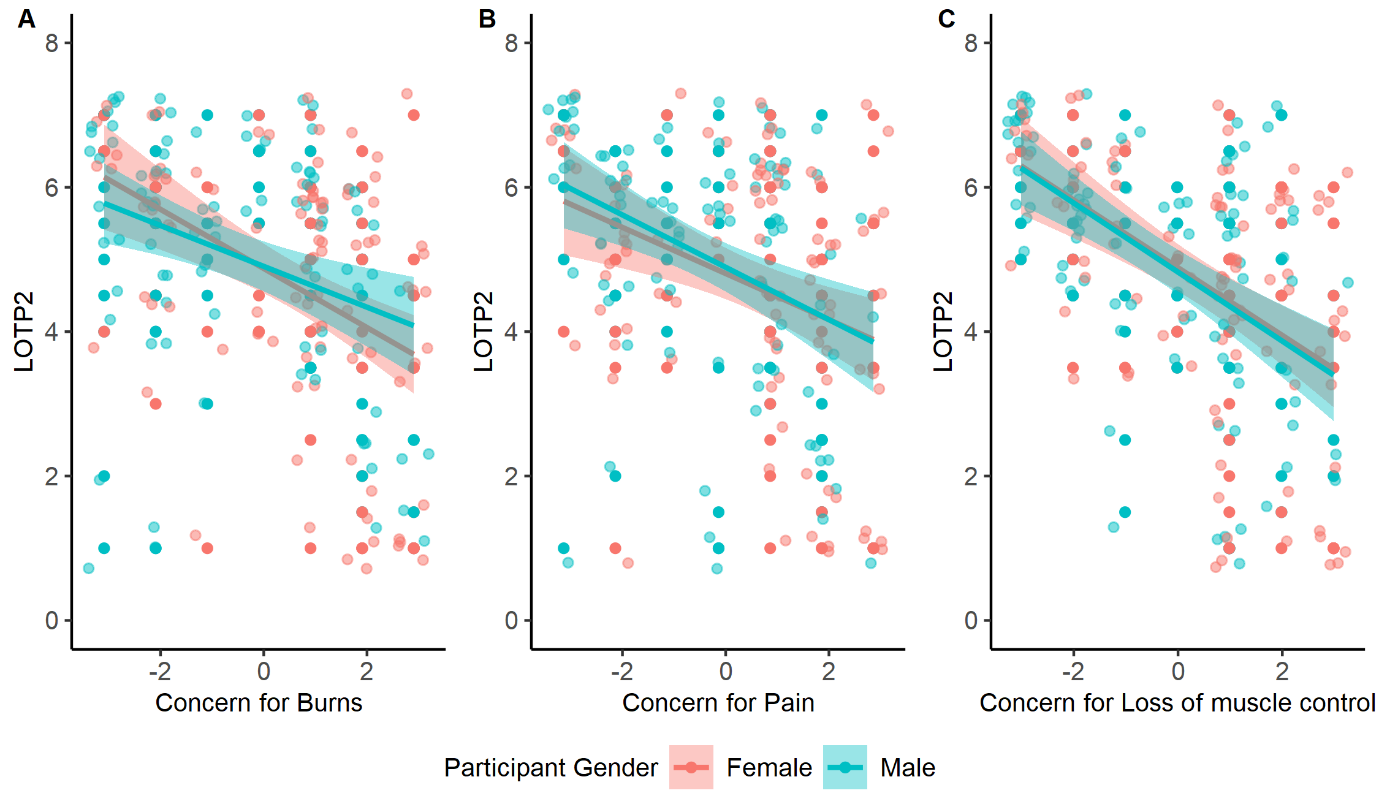


**S5.**

Results from multiple linear regression on 201 participants. The table shows significant results after the backwards elimination. F-values are from ANOVAs with Satterthwaite method for calculating degrees of freedom.

| Model | Term | Result |
| --- | --- | --- |
| LOTP1 | - | *R*^2^ = .08, *F*(2, 198) = 8.69, *p* < .001, adj. *R*^2^ = .07 |
|  | MAIA | β = -.32, 95% CI [.05, .60], *t*(195) = 2.30, *p* = .023 |
|  | Theoretical knowledge | β = .02, 95% CI [5.68e-03, .03], *t*(195) = 3.03, *p* = .003 |
| LOTP2 | - | *R*^2^ = .31, *F*(5, 195) = 17.74, *p* < .001, adj. *R*^2^ = .30 |
|  | Burns | β = -.14, 95% CI [-.26, -.02], *t*(195) = -2.34, *p* = .021 |
|  | LoC | β = -.27, 95% CI [-.40, -.14], *t*(195) = -4.03, *p* < .001 |
|  | MAIA | β = -.30, 95% CI [.02, .59], *t*(195) = 2.10, *p* = .037 |
|  | Theoretical knowledge | β = .02, 95% CI [6.75e-03, .03], *t*(195) = 3.29, *p* = .001 |
|  | NAQ avoidance | β = .18, 95% CI [.03, .34], *t*(195) = 2.38, *p* = .019 |

**S6.**

Complete survey presented to all participants via Qualtrics.

**fNMES Study 1 - SONA**

**Survey Flow**

**Block: Section 1 Information Sheet and Consent Form (3 Questions)**

**EmbeddedData**

**idValue will be set from Panel or URL.**

**Standard: Section 2 Demographics (5 Questions)**

**Standard: Section 2 Motivation to take part (3 Questions)**

**Standard: Section 3 Electrical stimulation experience (6 Questions)**

**Standard: Section 4 - Standardised Questionnaires (5 Questions)**

**Standard: fNMES 2 (11 Questions)**

**Standard: Debriefing and end (2 Questions)**

| Page Break |  |
| --- | --- |

**Start of Block: Section 1 Information Sheet and Consent Form**

Information Sheet  **Information Sheet**   My name is Themis Efthimiou and I am a PhD student in the Department of Psychology at the University of Essex. Together with my colleague’s Dr Sebastian Korb and Dr Paul Hanel, we would like to invite you to take part in this research study called “Attitudes towards receiving facial neuromuscular electrical stimulation”.    **Please take time to read the following information carefully.**   **Description**  Before you decide whether to take part, it is important for you to understand why the research is being done and what it will involve. The purpose of the study is to better understand your views and beliefs regarding facial neuromuscular electrical stimulation. You will be asked, what concerns you have about a hypothetical study, which uses facial neuromuscular electrical stimulation. Further, you will be asked to complete five questionnaires, related to personality, body awareness and image, and risk-taking.    **Duration** The study will take approximately 15 minutes.   **Eligibility Requirements**  You are eligible to take part in the study if you are 18 - 45 years old and are currently living in the UK.    **Withdrawal** Your participation is voluntary, and you will be free to withdraw from the project at any time without giving any reason and without penalty, by closing the browser. Incomplete responses will be considered withdrawn and deleted.  It will not be possible to delete your data after you have completed the experiment because the data are stored anonymously, so it will not be possible to identify your data.   **Data gathered** We will collect the following data, demographic information (age, gender, education level), concerns regarding a hypothetical study, and individual differences on different measures (described above) on five questionnaires**.** Your data will be fully anonymous so that it is not possible to identify you from our stored data. We are using your data to explore user concerns toward receiving facial neuromuscular electrical stimulation. Your data will be gathered by Qualtrics. Our legal basis for storing your consent form is that you have consented to it. The data controller is the University of Essex. Essex University's Data Protection Officer can be contacted on dpo@essex.ac.uk. Your anonymous data may be published in scientific journal articles and shared in permanent, publicly accessible archives accessible from any country.   **Funding** The research is funded by the Austrian-Science Fund.   **Ethics information** This study has been approved by the University of Essex Faculty of Science and Health Ethics Subcommittee, and had been given approval with the following Application ID: ERAMS reference:  ETH2021-0744.   **Concerns and complaints** If you have any concerns about any aspect of the study or you have a complaint, in the first instance please contact the Principal Investigators of the project (see contact details below). If you are still concerned or you think your complaint has not been addressed to your satisfaction, please contact the Director of Research in the Principal Investigator’s department (see below). If you are still not satisfied, please contact the University’s Research Governance and Planning Manager (Sarah Manning-Press).   Contact details  Principal investigators Dr Sebastian Korb (sebastian.korb@essex.ac.uk) Dr Paul Hanel (p.hanel@essex.ac.uk)   Director of Research, Dept of Psychology Prof Silke Paulmann (paulmann@essex.ac.uk)   University of Essex Research Governance and Planning Manager Sarah Manning-Press, Research & Enterprise Office, University of Essex, Wivenhoe Park, CO4 3SQ, Colchester. Email: sarahm@essex.ac.uk. Phone: 01206-873561

Consent Form **Attitudes towards receiving facial neuromuscular electrical stimulation**   **Researcher: Themis Efthimiou** **Principal Investigators: Dr Sebastian Korb and Dr Paul Hanel**
 **Consent Form**

|  | You need to agree with all responses to participate (1) |
| --- | --- |
| 1. I confirm that I have read and I understand the Information Sheet labelled ETH2021-0744. (1) |  |
| 2. I understand that no personal identifiable data will be collected. (2) |  |
| 3. I understand that my participation is voluntary and that I am free to withdraw from the project at any time without giving any reason and without penalty. I understand that my data cannot be deleted after I have completed the experiment because the data are stored anonymously, so it will not be possible to identify my data. (3) |  |
| 4. I understand that my fully anonymised data will be used for the research purposes outlined above and provided in detail at the end of the survey. (4) |  |
| 5. I understand that the anonymised data collected about me will be used to support other research in the future and may be made publicly available to benefit other researchers. (5) |  |
| 6. I agree to take part in the above study. (8) |  |

| Page Break |  |
| --- | --- |

Time_con Timing

First Click (1)

Last Click (2)

Page Submit (3)

Click Count (4)

**End of Block: Section 1 Information Sheet and Consent Form**

**Start of Block: Section 2 Demographics**

| 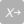 |
| --- |

Age What is your age (in years)?

▼ 18 (18) ... 45 (45)

| 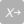 |
| --- |

Gender What is your gender?

- Male (1)
- Female (2)
- Other (3)
- Prefer not to say (4)

| 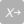 |
| --- |

Ethnicity How would you best describe your ethnic origin?

- White (1)
- Asian or Asian British (2)
- Black or Black British (3)
- Mixed (4)
- Other (5)

| 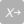 |
| --- |

Education What is the highest level of education you have completed?

- GCSEs or equivalent (typically exams at the age 16) (1)
- A-Levels or equivalent (typically exams at the age 18) (2)
- University undergraduate programme (3)
- University post-graduate programme (4)
- Doctoral degree (5)

*Display This Question:*

*If What is the highest level of education you have completed? = University undergraduate programme*

*Or What is the highest level of education you have completed? = University post-graduate programme*

*Or What is the highest level of education you have completed? = Doctoral degree*

Subject What subject did you study?

________________________________________________________________

**End of Block: Section 2 Demographics**

**Start of Block: Section 2 Motivation to take part**

Descriptor Imagine the following situation: 
You are invited to a research laboratory to take part in a paid (£10 per hour) psychological experiment about emotions. This study will use facial neuromuscular electrical stimulation, a safe and non-painful technique, to stimulate certain parts of your face with weak electrical impulses.

| 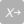 |
| --- |

LOTP How likely are you to take part in a study involving facial neuromuscular electrical stimulation?

- Extremely unlikely (1)
- Moderately unlikely (2)
- Slightly unlikely (3)
- Neither likely nor unlikely (4)
- Slightly likely (5)
- Moderately likely (6)
- Extremely likely (7)

SR_Concerns Please describe your concerns, if you were to receive facial neuromuscular electrical stimulation (one or two sentences)?

________________________________________________________________

________________________________________________________________

________________________________________________________________

________________________________________________________________

________________________________________________________________

**End of Block: Section 2 Motivation to take part**

**Start of Block: Section 3 Electrical stimulation experience**

Knowledge_ES Please rate your knowledge of electrical stimulation:

|  | Beginner | Expert |
| --- | --- | --- |

|  | 0 | 10 | 20 | 30 | 40 | 50 | 60 | 70 | 80 | 90 | 100 |
| --- | --- | --- | --- | --- | --- | --- | --- | --- | --- | --- | --- |

| Theoretical () | 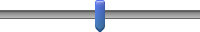 |
| --- | --- |
| Practical () | 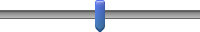 |

| Page Break |  |
| --- | --- |

| 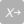 |
| --- |

Received_E-shock Have you ever received an accidental electrical shock?

- Yes (1)
- No (2)
- Unsure (3)

| Page Break |  |
| --- | --- |

*Display This Question:*

*If Have you ever received an accidental electrical shock? = Yes*

| 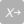 |
| --- |

shock_recency How recently did you receive this accidental shock?

- In the last week (1)
- In the last month (2)
- In the last 3 months (3)
- In the last 6 months (4)
- In the last year or longer (6)

| Page Break |  |
| --- | --- |

| 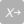 |
| --- |

Received_ES Have you ever received electrical stimulation, using a purpose built device?

- Yes (1)
- No (2)
- Unsure (3)

| Page Break |  |
| --- | --- |

*Display This Question:*

*If Have you ever received electrical stimulation, using a purpose built device? = Yes*

| 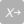 |
| --- |

Reason_electric What was the reason you received electrical stimulation

- Medical (1)
- Research purposes (2)
- Cognitive enhancement (3)
- Other (4)

*Display This Question:*

*If What was the reason you received electrical stimulation = Other*

ES_type Please specify the reason you received electrical stimulation

________________________________________________________________

**End of Block: Section 3 Electrical stimulation experience**

**Start of Block: Section 4 - Standardised Questionnaires**

| 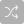 | 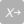 |
| --- | --- |

NFA   **Please indicate the extent to which you agree with the following statements:**
 

|  | Totally disagree (1) | (2) | (3) | Neither disagree nor agree (4) | (5) | (6) | Totally agree (7) |
| --- | --- | --- | --- | --- | --- | --- | --- |
| I feel that I need to experience strong emotions regularly. (1) |  |  |  |  |  |  |  |
| If I reflect on my past, I see that I tend to be afraid of feeling emotions. (2) |  |  |  |  |  |  |  |
| I find strong emotions overwhelming and therefore try to avoid them. (3) |  |  |  |  |  |  |  |
| Emotions help people get along in life. (4) |  |  |  |  |  |  |  |
| I think that it is important to explore my feelings. (5) |  |  |  |  |  |  |  |
| It is important for me to know how others are feeling. (6) |  |  |  |  |  |  |  |
| I would prefer not to experience either the lows or highs of emotions. (7) |  |  |  |  |  |  |  |
| I do not know how to handle my emotions, so I avoid them. (8) |  |  |  |  |  |  |  |
| Emotions are dangerous—they tend to get me into situations that I would rather avoid. (9) |  |  |  |  |  |  |  |
| It is important for me to be in touch with my emotions. (10) |  |  |  |  |  |  |  |
| It is important that you select totally agree (test item). (11) |  |  |  |  |  |  |  |

| Page Break |  |
| --- | --- |

| 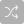 | 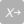 |
| --- | --- |

MAIA
**Below you will find a list of statements. Please indicate how often each statement applies to you generally in daily life.**

|  | Never (1) | (2) | (3) | (4) | Always (5) |
| --- | --- | --- | --- | --- | --- |
| When I feel physical pain, I become upset. (113) |  |  |  |  |  |
| I start to worry that something is wrong if I feel any discomfort. (114) |  |  |  |  |  |
| It is important that you select Never (test item) (115) |  |  |  |  |  |
| I can notice an unpleasant body sensation without worrying about it. (116) |  |  |  |  |  |
| I can stay calm and not worry when I have feelings of discomfort or pain. (117) |  |  |  |  |  |
| When I am in discomfort or pain I can’t get it out of my mind. (118) |  |  |  |  |  |

| Page Break |  |
| --- | --- |

| 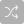 | 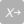 |
| --- | --- |

DOSPERT **For each of the following statements, please indicate the likelihood that you would engage in the described activity or behaviour if you were to find yourself in that situation. Provide a rating from Extremely Unlikely to Extremely Likely, using the following scale**

|  | Extremely unlikely (1) | Moderately unlikely (2) | Slightly unlikely (3) | Neither likely nor unlikely (4) | Slightly likely (5) | Moderately likely (6) | Extremely likely (7) |
| --- | --- | --- | --- | --- | --- | --- | --- |
| Drinking heavily at a social function. (1233) |  |  |  |  |  |  |  |
| Engaging in unprotected sex. (1236) |  |  |  |  |  |  |  |
| Driving a car without wearing a seat belt. (1237) |  |  |  |  |  |  |  |
| Riding a motorcycle without a helmet. (1238) |  |  |  |  |  |  |  |
| Sunbathing without sunscreen. (1270) |  |  |  |  |  |  |  |
| Walking home alone at night in an unsafe area of town. (1271) |  |  |  |  |  |  |  |

| Page Break |  |
| --- | --- |

| 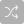 | 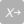 |
| --- | --- |

BICI **Please respond to each item by indicating how often you experience the described feelings or how often you perform the described behaviours.**

|  | Never (1) | Sometimes (2) | About half the time (3) | Most of the time (4) | Always (5) |
| --- | --- | --- | --- | --- | --- |
| I am dissatisfied with some aspect of my appearance. (1212) |  |  |  |  |  |
| I spend a significant amount of time checking my appearance in the mirror. (1213) |  |  |  |  |  |
| I feel others are speaking negatively of my appearance. (1214) |  |  |  |  |  |
| I am reluctant to engage in social activities when my appearance does not meet my satisfaction. (1215) |  |  |  |  |  |
| I feel there are certain aspects of my appearance that are extremely unattractive. (1216) |  |  |  |  |  |
| I buy cosmetic products to try to improve my appearance. (1217) |  |  |  |  |  |
| I seek reassurance from others about my appearance. (1218) |  |  |  |  |  |
| I feel there are certain aspects of my appearance I would like to change. (1219) |  |  |  |  |  |
| I am ashamed of some part of my body. (1220) |  |  |  |  |  |
| I compare my appearance to that of fashion models or others. (1221) |  |  |  |  |  |
| I try to camouflage certain flaws in my appearance. (1222) |  |  |  |  |  |
| I examine flaws in my appearance. (1223) |  |  |  |  |  |
| It is important that you select About half the item (test item). (1224) |  |  |  |  |  |
| I have bought clothing to hide a certain aspect of my appearance. (1225) |  |  |  |  |  |
| I feel others are more physically attractive than me. (1226) |  |  |  |  |  |
| I have considered consulting/consulted some sort of medical expert regarding flaws in my appearance. (1227) |  |  |  |  |  |
| I have been embarrassed to leave the house because of my appearance. (1228) |  |  |  |  |  |
| I fear that others will discover my flaws in appearance. (1229) |  |  |  |  |  |
| I have missed social activities because of my appearance. (1230) |  |  |  |  |  |
| I have avoided looking at my appearance in the mirror. (1231) |  |  |  |  |  |

| Page Break |  |
| --- | --- |

| 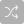 | 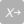 |
| --- | --- |

Big5 **Here are a number of characteristics that may or may not apply to you. Please indicate how much each statement accurately describes you.**

|  | Very inaccurate (1) | Moderately inaccurate (2) | Neither accurate nor inaccurate (3) | Moderately accurate (4) | Very accurate (5) |
| --- | --- | --- | --- | --- | --- |
| Have frequent mood swings. (16) |  |  |  |  |  |
| Have a vivid imagination. (17) |  |  |  |  |  |
| Am relaxed most of the time. (18) |  |  |  |  |  |
| Am not interested in abstract ideas. (19) |  |  |  |  |  |
| Get upset easily. (20) |  |  |  |  |  |
| Have difficulty understanding abstract ideas. (21) |  |  |  |  |  |
| Seldom feel blue. (22) |  |  |  |  |  |
| Do not have a good imagination. (23) |  |  |  |  |  |

| Page Break |  |
| --- | --- |

**End of Block: Section 4 - Standardised Questionnaires**

**Start of Block: fNMES 2**

Hypo_study   PLEASE READ CAREFULLY, as you will have to answer questions based on the text below.   We will now provide you with a more detailed description of what NMES is, and of how we plan to use it in a **hypothetical**, planned research experiment.    **What is NMES:** Neuromuscular Electric Stimulation (NMES), also known as TENS (Transcutaneous Electrical Nerve Stimulation), has been used for some time, especially in the field of physiotherapy and rehabilitation, including in the face. NMES can be used to induce muscle contractions, as it simulates the natural activation of a muscle.   **How do we intend to use NMES:** For the purpose of a planned experiment, four electrodes will be placed on your lower face. These are medically suitable, self-adhesive electrodes, which are disposed after each use. They are used for light electrical muscle stimulation, which takes place with medically certified and computer-controlled stimulators. You will receive NMES over specific facial muscles, mostly in short periods of approximately 1 second, and for a maximum total duration of 30 minutes. You will be compensated with £10 per hour. You are allowed to stop participating at any time.   **Side effects and Risks:** **Discomfort, Pain**: Most of the time, facial NMES will feel like a slight, non-painful tingling sensation. However, it can sometime feel unpleasant, and occasionally induce brief painful sensations. The level of pain/discomfort depends on many factors, including individual differences in pain sensitivity.   **Marks, burns**: The risk of getting skin burns exists, but is very low, as long as safety measures are followed, and stimulation limits are not exceeded. In some cases, however, light skin redness due to irritation can occur under the electrodes. This should disappear after few hours.   **Loss of control**: Be aware that your muscles will move without you controlling it. Your facial muscles may also feel tight and tired at the end of the experiment. Although not a health risk, these phenomena can be unpleasant for some people.    **Other**: For people with heart problems, a pacemaker or other implantable heart device, NMES may be dangerous and is not recommended. It is also not recommended during pregnancy.    PLEASE READ CAREFULLY, as you will have to answer questions based on the text above.

Time_hypo Timing

First Click (1)

Last Click (2)

Page Submit (3)

Click Count (4)

| Page Break |  |
| --- | --- |

| 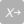 |
| --- |

NMES_understanding In the study just described, how many electrodes will be used?

- 2 electrodes (1)
- 4 electrodes (2)
- 6 electrodes (3)

| 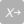 |
| --- |

NMES_understanding_2 In the study just described, in which part of the face is going to be stimulated?

- Upper (1)
- Lower (2)

| 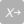 |
| --- |

NMES_understanding_3 Who cannot take part in the study just described?

- Pregnant people (1)
- Depressed individuals (2)
- People aged 25+ (3)

| Page Break |  |
| --- | --- |

| 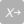 |
| --- |

LOTP_2 How likely are you to take part in a study involving facial neuromuscular electrical stimulation?

- Extremely unlikely (1)
- Moderately unlikely (2)
- Slightly unlikely (3)
- Neither likely nor unlikely (4)
- Slightly likely (5)
- Moderately likely (6)
- Extremely likely (7)

| Page Break |  |
| --- | --- |

| 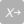 |
| --- |

LOTP_3 Do you intend to take part in a study involving facial neuromuscular electrical stimulation, if offered the possibility?

- I would never want to participate (1)
- - (2)
- - (3)
- I am undecided about participating (4)
- - (5)
- - (6)
- I absolutely want to participate (7)

| Page Break |  |
| --- | --- |

SR_concerns_2 What are your concerns about taking part in a study involving facial neuromuscular electrical stimulation?

________________________________________________________________

________________________________________________________________

________________________________________________________________

________________________________________________________________

________________________________________________________________

| Page Break |  |
| --- | --- |

| 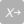 |
| --- |

Specific_concerns If I took part in a study involving facial neuromuscular electrical stimulation, I would be concerned about:

|  | Strongly disagree (1) | Disagree (2) | Somewhat disagree (3) | Neither agree nor disagree (4) | Somewhat agree (5) | Agree (6) | Strongly agree (7) |
| --- | --- | --- | --- | --- | --- | --- | --- |
| Getting burned (2) |  |  |  |  |  |  |  |
| Being in pain (3) |  |  |  |  |  |  |  |
| Losing control over my muscle (4) |  |  |  |  |  |  |  |

SR_concerns_3 Do you have any concerns that were not listed above?

________________________________________________________________

________________________________________________________________

________________________________________________________________

________________________________________________________________

________________________________________________________________

Time_concerns Timing

First Click (1)

Last Click (2)

Page Submit (3)

Click Count (4)

**End of Block: fNMES 2**

**Start of Block: Debriefing and end**

Q35 This is the end of the survey.
 Thank you very much for taking part, we greatly appreciate your contribution. This survey was interested in exploring the concerns participants have when considering whether to take part in a facial electrical stimulation study. Further, we aim to explore how one's willingness to take part differs across, gender, education level, personality, risk-taking, and body awareness.

 You are more than welcome to discuss your feelings about taking part in the survey below. If you have any questions/concerns or wish to find out more about the results of the study once the data has been analysed then send an email to: Themis Efthimiou (t.efthimiou@essex.ac.uk).

 If you have any further concerns, please feel free to contact the principal investigator, Dr Sebastian Korb (sebastian.korb@essex.ac.uk).

Debrief


Do you have any comments?

________________________________________________________________

________________________________________________________________

________________________________________________________________

________________________________________________________________

________________________________________________________________

**End of Block: Debriefing and end**
